# Niche Partitioning among Mesocarnivores in a Brazilian Wetland

**DOI:** 10.1371/journal.pone.0162893

**Published:** 2016-09-29

**Authors:** Rita de Cassia Bianchi, Natalie Olifiers, Matthew E. Gompper, Guilherme Mourão

**Affiliations:** 1 Departamento de Biologia Aplicada à Agropecuária, Universidade Estadual Paulista “Júlio de Mesquita Filho”, Jaboticabal, São Paulo, Brazil; 2 Laboratório de Biologia e Parasitologia de Mamíferos Silvestres Reservatórios, IOC/FIOCRUZ, Rio de Janeiro, Brazil; 3 Universidade Veiga de Almeida, Maracanã, Rio de Janeiro, Brazil; 4 School of Natural Resources, University of Missouri, Columbia, Missouri, United States of America; 5 Embrapa/Pantanal, Corumbá, Mato Grosso do Sul, Brazil; University of Regina, CANADA

## Abstract

We investigated the home range size, habitat selection, as well as the spatial and activity overlap, of four mid-sized carnivore species in the Central Pantanal, Mato Grosso do Sul, Brazil. From December 2005 to September 2008, seven crab-eating foxes *Cerdocyon thous*, seven brown-nosed coatis *Nasua nasua*, and six ocelots *Leopardus pardalis* were radio-collared and monitored. Camera trap data on these species were also collected for the crab-eating raccoon *Procyon cancrivorus*. We hypothesized that there would be large niche differentiation in preferred habitat-type or active period between generalist species with similar diet, and higher similarity in habitat-type or activity time between the generalist species (crab-eating foxes and coatis) and the more specialized ocelot. Individual home ranges were estimated using the utilization distribution index (UD– 95% fixed Kernel). With data obtained from radio-collared individuals, we evaluated habitat selection using compositional analysis. Median home range size of ocelots was 8 km^2^. The proportion of habitats within the home ranges of ocelots did not differ from the overall habitat proportion in the study area, but ocelots preferentially used forest within their home range. The median home range size of crab-eating foxes was 1.4 km^2^. Foxes showed second-order habitat selection and selected savanna over shrub-savanna vegetation. The median home range size for coati was 1.5 km^2^. Coati home ranges were located randomly in the study area. However, within their home range, coatis occurred more frequently in savanna than in other vegetation types. Among the four species, the overlap in activity period was the highest (87%) between ocelots and raccoons, with the least overlap occurring between the ocelot and coati (25%). We suggest that temporal segregation of carnivores was more important than spatial segregation, notably between the generalist coati, crab-eating fox and crab-eating raccoon.

## Introduction

Interspecific competition is an important mechanism structuring natural communities [1–3]. Through exploitation competition, a species can reduce the availability of a shared resource to another species, and through behavioral interference interactions, a species can alter the ability of other species to access such resources [4, 5]. Interspecific competition tends to restrict the range of habitats and resources a population uses because species normally differ in their ability to exploit habitat types and access resources [6]. Although the impact of competition is difficult to demonstrate in natural communities, studies suggest past and present competition among natural populations [6]. Many species of carnivorous mammals are adversely affected by other guild members, through both interference and exploitation competition [7–11]. Recent studies have documented effects of such interactions among carnivores, with important implications for the demographics of endangered species [11–14].

Closely related species are often similar morphologically, physiologically and behaviorally, and competition is likely to occur among such species when they are sympatric. Consequently, selection may be strong to render their ecological separation [15], creating three basic nonexclusive outcomes: (1) the species exploit different habitat types or microhabitats; (2) their diets differ; or (3) they are active at different times of day [6]. Thus, some sympatric and phylogenetically related species may alter habitat preferences or feeding habits to counteract competition [16–18] or may reduce competition by sequentially using shared resources [19].

Many studies discuss the importance of food preference [20–22], spatial partitioning [23, 24], or both [25], on interspecific competition, but time can also be a critical resource partitioned among species. Temporal segregation may facilitate the coexistence of species by reducing opportunities for interference competition, or the temporal overlap in resource consumption, if shared limited resources differ in period of availability. The latter is particularly true of competing predators whose prey show regular activity patterns [26].

In Neotropical carnivore communities there is evidence of niche partitioning among sympatric species [20, 27–29] but, in general, only one or two niche axes have been evaluated. Nonetheless, diet, behavior, and habitat use suggest high potential for interspecific competition in South American carnivoran assemblages. The ocelot *Leopardus pardalis*, for example, may reach high densities and exert considerable impact on small felid populations [30]. Similarly, in Neotropical canid assemblages, habitat use and feeding habits have been reported to show less overlap than does the active period [27], although other studies have demonstrated time as an important factor segregating canid species [28]. Thus it is possible that resource availability and opportunities for interaction with other carnivores can influence the extent of niche overlap.

The brown-nosed coati *Nasua nasua*, ocelot, crab-eating fox *Cerdocyon thous*, and crab-eating raccoon *Procyon cancrivorus* are medium-sized carnivores that are sympatric in most of their geographic distribution. They collectively form the primary biomass of mesocarnivores in most of South America. Although they are considered common and abundant in most biomes in which they occur, studies assessing their respective spatial distribution, habitat use, and activity patterns are scarce. In a previous study [31], we investigated food partitioning in foxes, coatis and ocelots, and showed large dietary overlap between the generalist fox and coati but a more specialized diet in the strictly carnivorous ocelot. We therefore hypothesize wide niche differentiation in habitat or active period among generalist species with similar diets, and higher similarity in habitat or active period when contrasting generalist species (crab-eating fox and coati) to the more specialized ocelots. In addition, despite the absence of information regarding feeding habits of crab-eating raccoons, we hypothesize a larger differentiation in habitat or active period of raccoons and coatis, because they are closely-related procyonids with similar morphology. To address these hypotheses, we investigated home range size, habitat use, and activity patterns of four abundant carnivores in the Brazilian Pantanal wetlands, one of the world’s largest remaining regions with an intact carnivore fauna.

## Methods

### Study area

The study was conducted in and around Nhumirim Ranch (18° 59'S, 56° 39'W), a 43-km^2^ research station of The Brazilian Agricultural Research Corporation (Embrapa) located in the Pantanal region of Mato Grosso do Sul. The Pantanal is a floodplain ecosystem with a tropical climate comprising wet (October to March) and dry (April to September) seasons. Human population density is <2 per km^2^, and the main economic activity is cattle ranching [32]. The study area is characterized by sandy soil with a mosaic vegetation of semi-deciduous forest, dispersed shrub vegetation, and seasonally flooded fields [33]. Permanent and temporary freshwater ponds and alkaline ponds occur throughout the area. We considered five habitat categories in the study area: forest, savanna, scrub-savanna, grassland, and ponds.

### Animal capture and handling

From December 2005 to September 2008, we captured ocelots, crab-eating foxes, and coatis up to four times per year. A grid of 36 trap stations spaced 500 m apart was set in the study area. At each node of the grid, a wire box live-trap (1m x 0.4m x 0.5m) was baited with bacon. Occasionally, traps were also placed out of the grid area to capture specific individuals. Traps were checked in the morning, closed, and reset late in the afternoon. Capture sessions were conducted every 3–4 months.

Captured individuals were anesthetized with Zoletil^@^50 (Virbac®; tiletamine hydrochloride and zolazepan hydrochloride, 10 mg/kg), marked with colored ear-tags, fitted with a subcutaneous transponder (Transponder ISO FDX-B, 134, 2 Khz, AnimalTAG^®^), measured, sexed, and weighed. A subset of animals was equipped with VHS radio-collars (ATS^®^or Telonics^®^). The reproductive status of females (apparent or non-enlarged nipples) was recorded, and tooth eruption and wear were used to estimate age [34, 35]. Animals were monitored until recovery from anesthesia and then released at the site of capture. After the end of the study, we were able to recapture almost all animals to remove their radio-collars. The exceptions were those animals that had dispersed out of telemetry and tracking range or possibly those animals who remained at the study site, but were not trapped and their radio-collars stopped functioning such that their ultimate whereabouts were unknown. This study and handling procedures were approved by the Brazilian Government Institute for Wildlife and Natural Resources Care (IBAMA, first license #183/2005 –CGFAU/LIC; last license #11772–2) and the University of Missouri Animal Care and Use Committee (protocol #4459).

### Camera trapping

**Fig 1:**
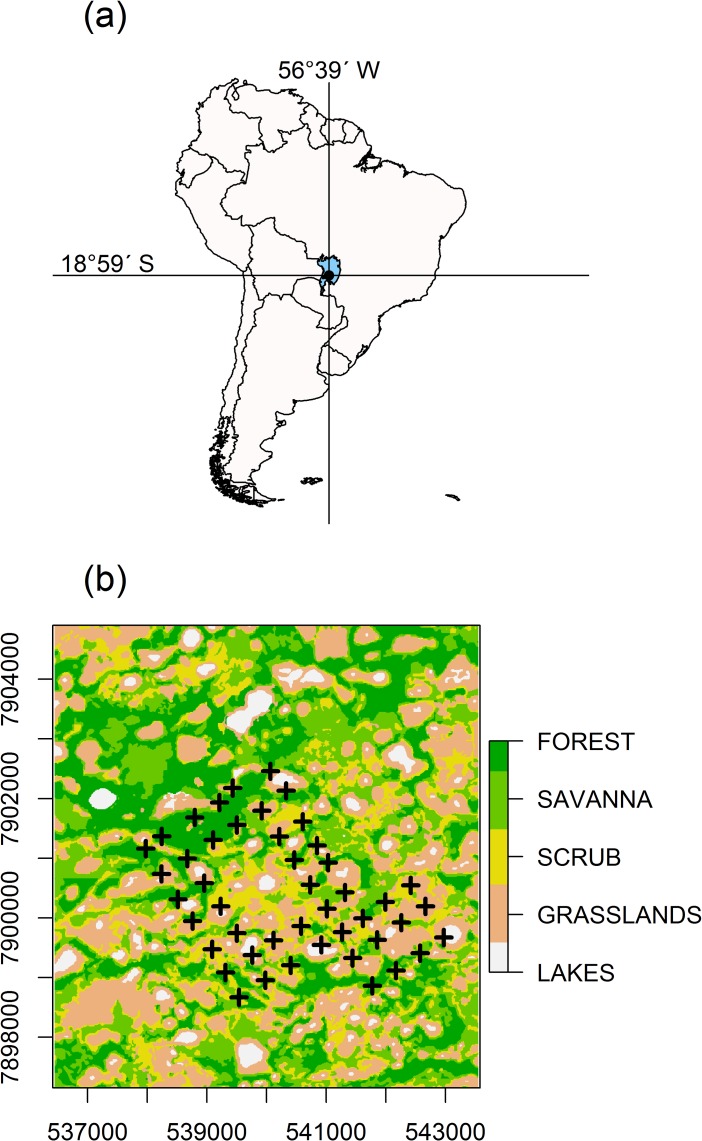
**(a) Map of South America showing the Brazilian Pantanal (in blue).** The lines cross at the study area (Nhumirim Ranch and neighboring areas); (b) thematic map of the study area showing the grid of camera-traps. The coordinates of the thematic map are shown as meters in zone 21k of the Universal Transverse Mercator system.

Twelve camera traps (Tigrinus^®^) were used as an additional method of recording the presence of marked individuals to aid in the assessment of habitat use, activity, and the home range size of radio-collared animals. Camera traps were installed at capture stations of the grid every three months. The trapping grid was divided into three blocks of 12 stations, and cameras were placed in each block for six consecutive days, after which they were moved to a second block of the grid. This procedure was repeated three times, until each of the 36 grid nodes had been sampled for six consecutive days (16 to 20 days total sampling time). Thereafter, the camera traps were placed next to roads and trails, usually out of the grid (Fig 1). Cameras were active 24 hours, with a 10 second-interval between photographs. We checked cameras every 1–2 days for bait and attractant replacement (bacon and *Lynx rufus* urine).

### Radio-telemetry and home-range calculation

Radio-locations were obtained through homing, which consisted of following the signal from a radio transmitter until the animal or the transmitter signal could be heard without the aid of the antenna. This technique reduces the error in assessment of habitat use analyses that usually arises when using locations from antenna angulation without a fixed base, held by a single person, especially in an area with such heterogeneous vegetation. Radio-collared animals were tracked using a radio receiver (TR-4 Telonics^®^) and a 3-element Yagi antenna, and animal locations were recorded using a portable GPS. At each location, we recorded habitat-type and time of location, whether the animal was active, and the presence of nearby conspecifics (coati bands, family groups, breeding pairs). Locations were obtained at various times throughout the day and night. Special emphasis was given to identifying the nesting locations of female coatis. Female coatis leave the band when pregnant and construct an arboreal nest where they give birth and raise their young for about 4 weeks [34].

Each animal was localized once a day in different hours. We located all animals during the day and night, but total monitoring time varied between individuals (from 85 to 873 days; Table 1) mainly due to the functionality of radio-collars or because some animals died. The visual inspection of the frequency distribution of home range sizes suggested non-normality. Therefore, we used the median as well as minimum and maximum home range sizes as summary statistics. We estimated utilization distributions (UD) for individual animals for which ≥20 locations were obtained [36, 37]. This threshold could be considered low [38] but just one ocelot and one coati had such small number of locations and their estimated UD were of the same magnitude as those of conspecifics with larger number of locations. Because kernel estimates are sensitive to the density of locations, i.e., can become smaller with increased locations, we preferred to use the minimum convex polygon (MPC 100%) to construct the individual cumulative curves of home range size as a functions of increasing sampling effort. We calculated UDs using fixed kernel estimators with the KDE function in Matlab (The Mathworks Inc, Natick, MA USA;[39]). Kernel size or bandwidth was selected using the plug-in method [40, 41]. We excluded the outer 5% of the interpolated UD estimate using Hawth’s tools in ArcGIS v. 9.1 (Environmental Systems Research Institute, Redlands, CA, USA) to reduce potential bias in home range size estimates resulting from low use areas at the tails of the UD.

**Table 1:** Estimates of home range size of ocelots, crab-eating foxes, and brown-nosed coatis radio-tracked from December 2005 to May 2008 in Nhumirim Ranch, Pantanal, Brazil. UD = Utilization distribution calculated from 95% fixed kernel estimates.

Species	ID	Sex	Locations	UD (km^2^)	% locations		Days of monitoring
					Day	Night	
Ocelot	LP1	Female	77	7.0	53.2	46.8	618
	LP2	Female	52	4.5	50.0	50.0	613
	LP3	Female	42	8.0	73.8	26.2	348
	LP4	Female	21	8.8	52.4	47.6	85
	LP5	Male	59	16.1	50.8	49.2	502
	LP6	Male	34	3.8	61.8	38.2	169
Crab-eating fox	CT1	Female	53	2.3	37.7	62.3	350
	CT2	Male	86	1.2	62.1	37.9	472
	CT3	Male	83	1.4	48.6	51.4	774
	CT4	Female	93	2.2	50.4	49.6	427
	CT5	Female	79	1.7	62.7	37.3	473
	CT6	Female	65	1.0	58.1	41.9	471
	CT7	Male	45	0.9	56.8	43.2	298
Brown-nosed coati	NN1	Female	66	3.4	59.1	40.9	823
	NN2	Female	57	3.6	66.7	33.3	287
	NN3	Female	51	1.5	82.4	17.6	486
	NN4	Female	39	1.5	61.5	38.5	171
	NN5	Male	33	1.2	51.5	48.5	354
	NN6	Male	55	0.6	76.4	23.6	472
	NN7	Male	19	1.4	47.4	52.6	155

We used two indices to estimate home range overlap [42]. The first gave the probability of animal *j* being located in animals *i’*s home range (PHR) and the second represented the joint distribution of use of the two animals, the utilization distribution overlap index (UDOI), under the assumption that the animals use space independent of one another. A UDOI of 0 means that the two home ranges do not overlap. If both UDs are uniformly distributed and have 100% overlap, UDOI = 1. When the two UDs are not uniformly distributed and have a high degree of overlap, UDOI values can be >1 [42].

### Habitat selection analysis

Habitat selection by species was analyzed using Type II and III designs in which each individual is a sampling unit, and habitat use and availability are compared for each individual [43, 44]. For these analyses, we used information obtained from radio-collared animals. We utilized the UD estimated by 95% fixed kernels, deleting the 5% using Hawth's tools in ArcGIS 9.1 (Environmental Systems Research Institute, Redlands, CA, USA). For each species, the study area was considered to be the polygon created with the home range of all radio-tracked individuals, reducing the subjectivity of what is considered be the study area [45]. For analyses of Type II habitat selection, habitat availability was assessed throughout the study area, as defined by the minimum convex polygon around the home ranges of all animals of each species, and Type III was conducted within the home range of each individual. Thus, the Type II and III designs reflect second and third order habitat selection, respectively [46]. We used the map with vegetation classifications modified from Rodela [33] to estimate the availability of habitats.

We used Resource Selection for Windows (RSW) to conduct the compositional analysis [47]. The compositional analysis uses multivariate analysis of variance (MANOVA) models to analyze log-ratios for comparison of utilization and availability of habitats [48]. In a compositional analysis, use of each habitat *Ui* is expressed relative to each of the other habitats *U*_*j*_, as a log ratio ln*(U*_*i*_/*U*_*j*_), with availability being the equivalent ln(*V*_*i*_/*V*_*j*_). No difference between ln(*U*_*i*_/*U*_*j*_)-ln(*V*_*i*_/*V*_*j*_) indicates that animals have a similar association for each habitat pair *i* and *j*. Differences larger than zero indicate a selection of one habitat over another. Using an assumption of normality, Wilk’s λ can be calculated for the resulting matrix of pairwise values for an overall test of non-random use [43, 49]. Proportional availability of each habitat in the study area is compared to proportional availability of habitats in home ranges (Type II) and proportional availability of habitats in the home range is compared to proportions of radio locations for each individual (Type III) [48].

All habitats were exploited in proportions larger than zero; therefore it was not necessary to replace 0 with 0.01%, a commonly used procedure that can lead to increased probability of type I error [50]. All statistical analyses were performed using program SYSTAT 11 for Windows [51].

### Activity patterns

We used the time at which animals were photographed to evaluate the activity pattern. Because the study occurred across seasons, we adjusted the times when the animals were active between sunrise and sunset. For this, we used the daily times of sunrise and sunset at the study site in the Sunrise/Sunset/Sun Angle Calculator tool available at http://www.hia-iha.nrc-cnrc.gc.ca/sunrise_adv_e.html. The duration corresponding to a set time of day was calculated as the duration of the light period divided by 12 and the set time of night as the duration of the dark period divided by 12.

We used a conditional circular kernel density function to estimate overlap in activity patterns [52]. This circular kernel had the same features as the home range kernel estimator, including a smoothing parameter and a conditional density isopleth, defined as the threshold of probability that specifies the section of the function that accounts for a given proportion of the entire probability function [52]. We estimated 95% and 50% overlap of active periods of the studied species.

## Results

### Sample and home range sizes

**Fig 2:**
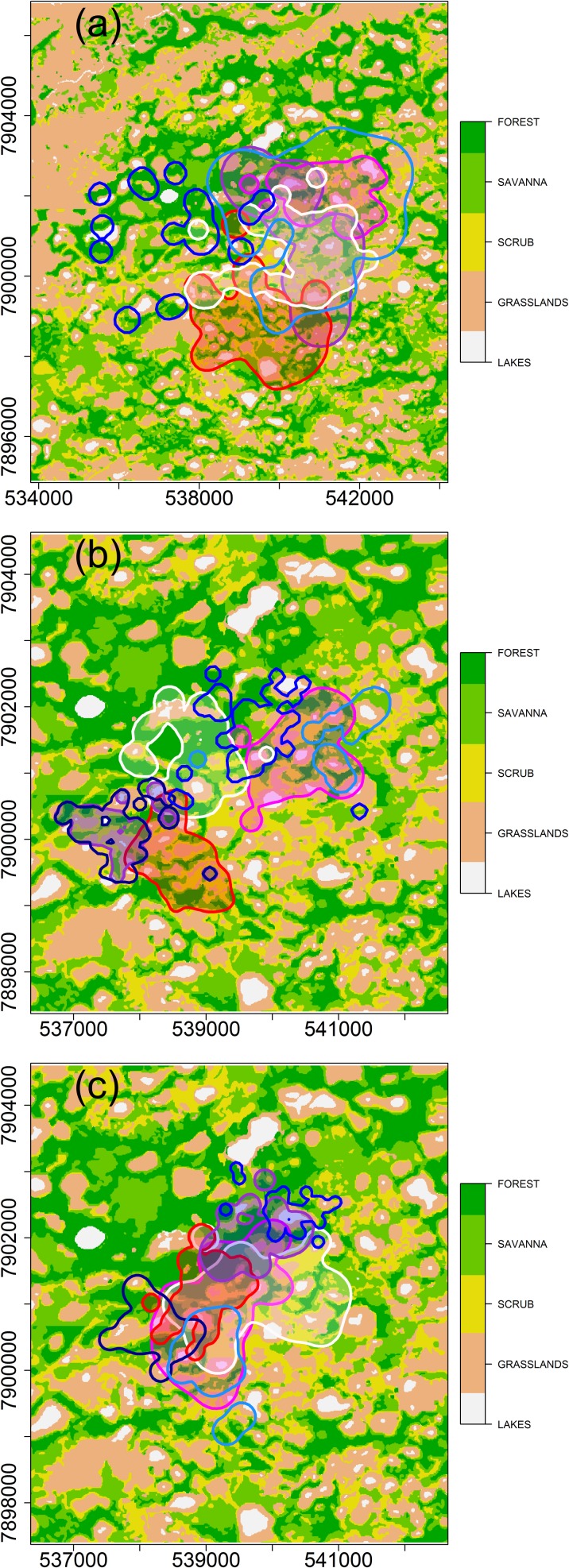
**Thematic maps of the Nhumirim ranch and neighboring areas showing the home range contours (estimated by 95% fixed kernels) of**: (a) three female and two male ocelots (male home ranges are shown in blue shades); (b) four female and three male crab-eating foxes (male home ranges are shown in shades of blue); (c) four female and three male coatis (male home range are shown in blue shades). The coordinates of thematic maps are shown as meters in zone 21k of the Universal Transverse Mercator system. Data were collected from December 2005 to September 2008.

**Fig 3:**
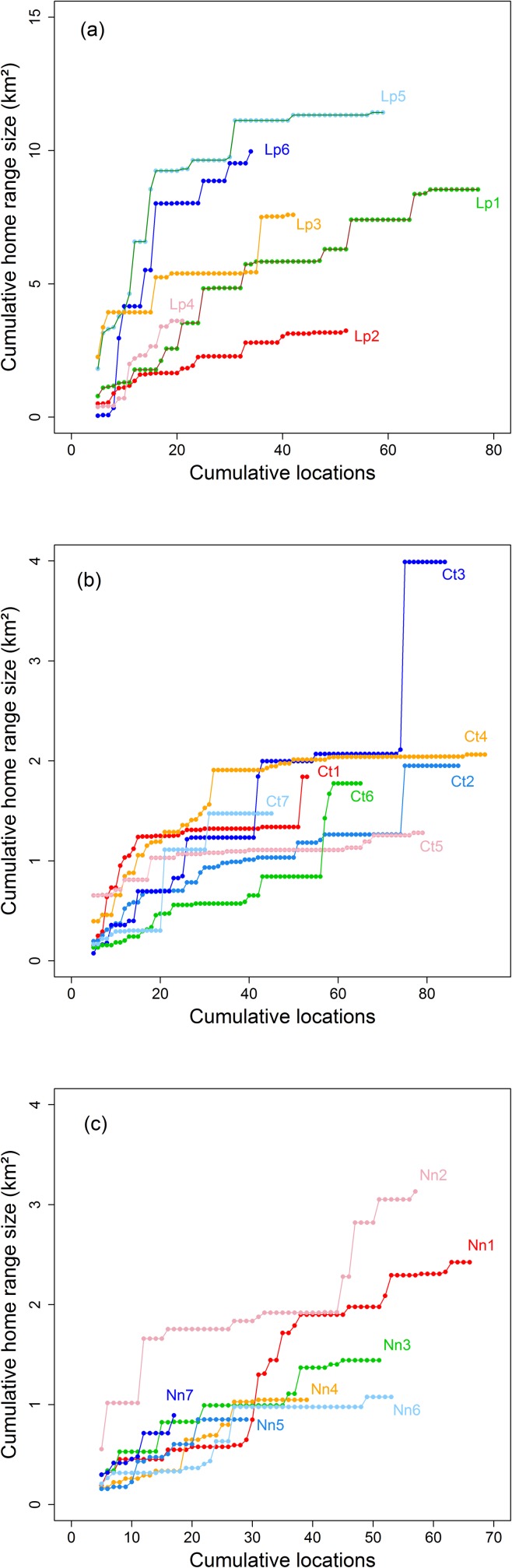
Cumulative curves of home range size (minimum convex polygon 100%) by number of locations of (a) ocelots, (b) crab-eating foxes and (c) coatis.

Fifteen individual ocelots were captured 30 times in total, 76 crab-eating foxes were captured 217 times, and 103 brown-nosed coatis were captured 99 times. Two crab-eating raccoons were captured, but few locations were obtained for these individuals. Thirteen ocelots, eight crab-eating foxes, and 13 brown-nosed coatis were fitted with radio-collars, of which six ocelots, seven foxes, and seven coatis were found in ≥ 20 locations and used in subsequent analyses. The median home range estimate was 8.0 km^2^ (3.8–6.1 km^2^) for ocelots (n = 6), 1.4 km^2^ (0.9–23 km^2^) for crab-eating foxes (n = 7), and 1.5 km^2^ (0.6–3.4 km^2^) for coatis (n = 7; Table 1; Fig 2). In most cases, the number of locations was sufficient to estimate home range size of the all individual (Fig 3).

In general, there was substantial home range overlap among species. The probability of home-range overlap (PHR) indicated 70% and 61% chance of an ocelot being found in the home range of a fox or coati, respectively. The probability of a crab-eating fox or a coati being found in the home range of an ocelot was close to 100% (Table 2). The joint distribution of use (UDOI) between the specialized ocelot and the generalist carnivores was lower than the UDOI among the generalists.

**Table 2:** Percentage of home range overlap (PHR) among the studied species, from December 2005 to May 2008 in Nhumirim Ranch, Pantanal, Brazil. Values of PHR in columns represent the probability of these animals being in the UD of the animals in rows. The joint distribution of use (UDOI) between the studied species is shown in parentheses.

Species	Ocelot	Crab-eating fox	Brown-nosed coati
**Ocelot**	95.0 (1.27)	96.2 (0.56)	99.5 (0.46)
**Crab-eating fox**	70.0	95.0 (1.29)	95.0 (0.79)
**Brown-nosed coati**	61.2	80.8	95.0 (1.38)

### Habitat selection

Ocelots were not selective in the location of their home ranges within the study area (Type II habitat selection; λ = 0.340; χ²_(4)_ = 6.473; p = 0.167). However, ocelots used habitat types within their home range (Type III habitat selection) in a proportion different than would be expected by chance (λ = 0.111; χ²_(4)_ = 13.206; p = 0.01), selecting forest over other habitat types (Table 3).

**Table 3:** Matrix and habitat ranking of Type III (Third order) resource selection by ocelots from December 2005 to May 2008 in Nhumirim Ranch, Pantanal, Brazil. Higher ranks represent higher levels of selection. P-values are given in parentheses.

Habitat type	Lakes	Savanna	Grasslands	Scrub savanna	Ranking
**Forest**	1.218 (0.27)	0.986 (0.37)	1.076 (0.33)	1.081 (0.33)	4
**Lakes**	.	0.900 (0.41)	1.005 (0.36)	1.015 (0.35)	3
**Savannah**		.	0.816 (0.45)	2.280 (0.07)	2
**Grasslands**			-	1.021 (0.35)	1
**Scrub savanna**				.	0

In contrast, the home ranges of crab-eating foxes were more likely to be located in areas of savanna (λ = 0.092; χ^2^_(4)_ = 16.635; p = 0.002), as opposed to forest, relative to its availability in the study area (p = 0.05; Table 4). However, foxes did not select specific habitat types within their home range (λ = 0.650 χ²_(4)_ = 3.021; p = 0.55).

**Table 4:** Matrix and habitat ranking of Type II (second order) resource selection by crab-eating foxes captured from December 2005 to May 2008 at Nhumirim Ranch, Pantanal, Brazil. Higher ranks represent higher levels of selection. P-values are given in parentheses.

Habitat types	Grasslands	Scrub savanna	Lakes	Forest	Ranking
**Savanna**	0.889 (0.41)	0.993 (0.36)	0.999 (0.35)	2.434 (0.05)	4
**Grasslands**	-	0.044 (0.96)	0.874 (0.41)	0.981 (0.36)	3
**Scrub savanna**		-	0.675 (0.52)	1.049 (0.33)	2
**Lakes**			-	0.134 (0.89)	1
**Forest**				-	0

Coatis did not show selection for home range location within the study area (λ = 0.698; χ²_(4)_ = 2.515; p = 0.642). However, within their home ranges, coatis selected savanna over other habitat types (λ = 0.192; χ²_(4)_ = 11.539; p = 0.021) (Table 5). All 39 nesting locations of seven monitored female coatis from four groups were within, or at the borders of, forest patches.

**Table 5:** Matrix and habitat ranking of Type III (third order) resource selection by coatis from December 2005 to May 2008 in Nhumirim Ranch, Pantanal, Brazil. Higher ranks represent higher levels of selection. P-values are given in parentheses.

Habitat type	Grasslands	Forest	Lakes	Scrub savanna	Ranking
**Savanna**	0.496 (0.64)	1.028 (0.34)	1.223 (0.27)	1.008 (0.42)	4
**Grasslands**	-	0.582 (0.58)	1.398 (0.21)	1.010 (0.35)	3
**Forest**		-	0.536 (0.61)	0.869 (0.42)	2
**Lakes**			-	0.521 (0.62)	1

### Activity patterns

From February 2007 to February 2009, with a capture effort of 2238 camera trap-days, we obtained 1773 photos of nine species of carnivores. The species with the highest capture success was the crab-eating fox (66% of records; n = 1176), followed by the brown-nosed coati (24%; n = 419), the crab-eating raccoon (4.5%; n = 77), and the ocelot (3.5%; n = 68).

Foxes showed crepuscular-nocturnal activity that peaked between 17.00 h and 21.00 h. During the day, foxes were recorded at low frequency, and no records occurred between 11.00 h and 12.00 h. Coatis were essentially diurnal, with night records occurring at low frequency. Ocelots and raccoons were more active at night. Daytime records of ocelots were scarce (n = 5) and were absent for raccoons.

**Fig 4:**
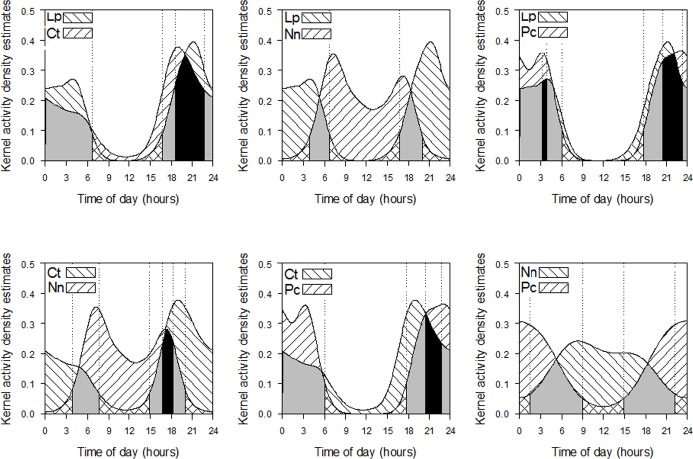
Overlap of active period among carnivore species. Gray = 95% overlap, black = 50% overlap. Lp = *Leopardus pardalis* (ocelot), Ct = *Cerdocyon thous* (crab-eating fox), Nn = *Nasua nasua* (brown-nosed coati), Pc = *Procyon cancrivorus* (crab-eating raccoon).

Among the four species, the overlap in active period of ocelots and raccoons was highest (87%), with the least overlap occurring between ocelot and coati (25%) (Fig 4). The overlap in the active period of ocelots and foxes (80%), and of foxes and raccoons (70%), was higher than the overlap between foxes and coatis (38%) and between raccoons and coatis (45%) (Fig 4).

## Discussion

Space use is based on behavioral and physiological/biological requirements [53], and a clear relationship usually exists between a species home range size and its body mass and diet, with the relationship mediated by landscape productivity [54–57]. Our estimates of home range size for ocelots were about twice those previously reported in the same area [58], but nonetheless low compared with the mean values reported from other sites [59]. For example, the median number of locations for ocelots in other areas was 110, ranging from 10 to 758 [58, 60–69]. While some differences in home range estimates may be attributed in part to the number of locations, the duration of monitoring or the estimator used [70], the small home ranges observed in the present study may reflect the high resource availability of the Pantanal landscape. The home ranges of the omnivorous crab-eating fox were smaller than those of ocelots, similar to those previously estimated for the region (1.2 km^2^) [58], and on the low end of the range of reported estimates in other regions [61, 71–73]. There is little information regarding the spatial ecology of the brown-nosed coati, despite its high abundance and diurnal activity pattern. In this study, size estimates of coati home ranges were similar to those observed for foxes.

Environmental spatial complexity may promote coexistence when both specialist and generalist species are present [74]. Therefore, heterogeneous landscapes such as that of the Pantanal may facilitate coexistence of species of the same trophic level [23]. Forested habitats are important for ocelots in the Pantanal, although ocelots were not observed to select a particular habitat type at the second order. Ocelots in southern Texas have been reported to prefer dense forest cover and to move primarily in forest corridors, avoiding open areas [64]. However, most of the open habitats in that area are characterized by high anthropogenic modification [75]. Although legally protected, ocelot populations continue to be threatened by deforestation and fragmentation [76, 77], and the maintenance of forest habitats can be critical to the conservation of this species [78, 79] throughout its distribution.

Crab-eating foxes selected savanna as home ranges, but used all habitats within their home range randomly. In other studies in Central Brazil, crab-eating foxes selected grasslands and savanna [80] or used all available habitat types in proportion to their availability [27]. This species seems to have considerable flexibility in the use of habitat and can sometimes benefit from anthropogenic disturbance [81].

Within their home ranges, coatis were more frequently found in savanna vegetation but require forest for building their reproductive nests. During the reproductive period, no radio-tracked female was located outside forest patches. It is likely that predation pressure on adult coatis was small in the study area, since no radio-tracked animal was preyed upon in the study area, and a separate study found coati hair in only one of 46 ocelot scats [31]. The use of forested habitat may reduce predation upon offspring during a critical period.

While a dichotomous division of diurnal and nocturnal animals is widely recognized, closer examination of temporal niches for mammals reveals a continuum in activity patterns, with diurnal and nocturnal at opposite extremes [82]. In this study, the species showed a gradient of activity, with coatis being crepuscular-diurnal, crab-eating foxes being vesperal-nocturnal, ocelots being vesperal-nocturnal to nocturnal, and crab-eating raccoons being strictly nocturnal. There is no consensus on how temporal partitioning enables the coexistence of species of similar size and habits. In Venezuela, activity pattern was not found to be relevant in the ecological separation of five carnivores, including ocelot and crab-eating fox, while diet was clearly different among the species [61]. Studies of the crab-eating fox and Pampas fox *Pseudalopex gymnocercus* revealed no differences in activity patterns but, in these studies, the species were studied in different regions of the park [83, 84]. Separate active periods have been reported to facilitate the coexistence of these species, and the Pampas fox was observed to shift its active period in the presence of the crab-eating fox [85]. In a study of three sympatric canids, diet and habitat use were reported to be more important in niche separation than activity time [27]. In Belize, studies of the carnivore community have not reported habitat preference or partitioning among species, even similar-sized species such as the puma *Puma concolor* and jaguar *Panthera onca*, or potential intraguild predators such as the ocelot, jaguar, and puma [24]. Data of the present study indicated time as a more important variable in the segregation of carnivores than habitat type, especially among coatis and potential competitors for food and habitat, as is the case of the crab-eating fox and the crab-eating raccoon [31]. The results are consistent with our hypothesis of a large niche differentiation in habitat or active period between the generalist species with similar diet, crab-eating fox and coati, and greater similarity in habitat or active period between those species and the more specialized ocelots. The more similar in diet the species were, the more they differed in activity patterns and habitat selection at the studied scales.

Although difference in diet may be an important mechanism for avoiding competition, the active period can be even more important, as was suggested in this study. This is not because the resource will be renewed in a few hours (like nectar production, for example) but rather to avoid interference competition. Interference competition can be an important selective force among carnivores. Some species use suboptimal habitats with low prey density to avoid competitors or predators [11, 86]. In Neotropical felid assemblages, margays *Leopardus wiedii* and oncillas *L*. *guttulus* have been reported more abundant in the most degraded areas, probably due to lack of competition from ocelots and jaguarondi in those areas [29]. Although agonistic interactions among Neotropical carnivores are difficult to demonstrate, some intraguild predation has been reported, mainly among felids [30]. It is possible that the ocelot is a dominant competitor in the study area and has a strong impact on other felid species such as the jaguarondi and the pampas cat, few of which were caught on camera in the area, and when caught the occurrences were solely during the day [87]. It has been demonstrated that the presence of ocelots has a negative effect on the population density of other small felid species [59]. Questions of how a single generalist or more abundant species can impact rare or threatened species are important management issues when considering the conservation of rare and endangered species [10, 11, 88].

In the Pantanal, ocelots can more directly affect other felids species than generalist carnivores such as coatis and crab-eating foxes. Competition between coatis and crab-eating foxes may be avoided by distinct temporal activities or by differential habitat use at spatial scales not evaluated in this study. Although both species had the savanna and grasslands as important habitats, the way the two species exploit these habitats differs. For instance, coatis are scansorial and are the only species adapted to use the vertical stratum systematically for foraging [31] and shelter [34]. In addition, the olfactory abilities of coatis allow this species to exploit resources such as underground invertebrates more efficiently than crab-eating fox.
